# An unusual presentation of metastatic prostate cancer in a 44‐year‐old man: A case report and review of the literature

**DOI:** 10.1002/ccr3.8447

**Published:** 2024-01-28

**Authors:** Homa Taheri, Pouya Ebrahimi, Pedram Nazari, Amirhosein Kefayat, Abbas Mahdavian

**Affiliations:** ^1^ Cedars‐Sinai Cardiology Department Beverly Hills California USA; ^2^ Ahvaz Jundishapur University of Medical Sciences Ahvaz Iran; ^3^ Cancer Research Center Ahvaz Jundishapur University of Medical Sciences Ahvaz Iran; ^4^ Oncology Department Isfahan University of Medical Sciences Isfahan Iran; ^5^ Urology Department Ahvaz Jundishapur University of Medical Sciences Ahvaz Iran

**Keywords:** cancer, case report, early‐onset, metastasis, prostate

## Abstract

Prostate cancer is one of the two most common non‐cutaneous cancers in men. Its presentation might be with unusual symptoms and cause the wrong initial diagnosis. This case report discusses a rare neurologic manifestation of advanced metastatic cancer in a low‐risk man. He had been receiving treatment for multiple sclerosis incorrectly due to unusual manifestations such as claudication and pelvic, leg, and shoulder pain. The patient underwent a whole‐body bone scan and then a transrectal ultrasound‐guided biopsy, which confirmed metastatic prostate cancer with a Gleason score between 7/10 and 10/10 in all samples. Following treatment with chemotherapeutic injections (docetaxel), luteinizing hormone‐releasing hormone (LHRH) analogous (Zoladex), and testosterone‐suppressing tablets (abiraterone), the disease has been under control and prostate‐specific antigen (PSA) level has decreased significantly. The most common sites of metastasis are regional lymph nodes, bones, and lungs. However, there are reports about the spread of this type of cancer to other parts of the body. Although most patients are diagnosed when the tumor is localized to the prostate, in about 25% of patients, the disease is diagnosed when metastasis has occurred. Some markers can assist physicians in the diagnosis of this disease, such as the Prostate Health Index and the 4 K score.

**Key Clinical Message:**

The diagnosis of prostate cancer should be considered in all age ranges of adult men. The long‐distance metastasis might cause unusual presentations of the disease, such as neurologic, musculoskeletal, and dermatologic symptoms and signs far from the origin of the cancer, before genitourinary manifestations. It is crucial to keep the diagnosis of prostate cancer in mind for men with suggestive signs and symptoms that are not usually detected in this disease.

## Graphical abstract

An unusual presentation of an invasive metastatic prostate cancer

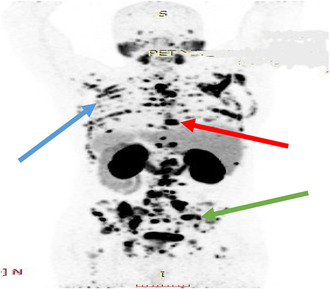

## 1 INTRODUCTION

Prostate cancer is one of the two most common non‐cutaneous cancers in men and the fifth main cause of death among both genders in the world.^1^ Although the risk of occurrence of this cancer is correlated with aging, the diagnosis of early prostate cancer has had an increasing trend during the past decade, which can be attributed to the emergence of screening methods.^2^ Screening tests such as Prostate Specific Antigen (PSA), have decreased the percentage of metastatic cancer and this cancer's mortality rate.^3^ On the contrary, the low specificity of this test has caused a large number of unnecessary biopsies. That's the reason new models of screening have been evaluated and considered recently.^4^ These efforts have resulted in the introduction of new markers such as the Prostate Health Index (PHI) and the 4K Score. These markers have revealed promising performance and accuracy in recent studies.^4^, ^5^

Bone is a preferential site of metastasis in prostate cancer metastasis, with synchronous involvement most commonly in the lungs and liver.^6^ There have also been reports of unusual presentations of metastatic prostate cancer such as neurologic, dermatologic, musculoskeletal, and rare urologic manifestations.^7^, ^8^, ^9^, ^10^, ^11^ However, this case seems to be unique due to the neurologic and musculoskeletal presentation of the disease far from the pelvis in a relatively young patient, which resulted in the misdiagnosis of multiple sclerosis and the wrong treatment for more than 5 years.

## 2 CASE PRESENTATION

A 44‐year‐old male came to our tertiary outpatient urology clinic with complaints of hip pain and the inability to bear weight on the lower limbs for several months, in addition to right shoulder pain. There was no history of trauma or other systemic symptoms. He had been treated for the diagnosis of Multiple Sclerosis for more than 5 years. The pain was not responsive to non‐steroidal anti‐inflammatory drugs (NSAIDs) and other painkillers. The physical examination was abnormal and revealed muscle weakness and lower limb claudication. A transrectal prostate examination revealed an asymmetrically enlarged prostate. The PSA level was 258.3 ng/mL (normal range:0–4 ng/mL). Due to a rare manifestation of prostate cancer at this age, the patient was diagnosed with acute prostatitis and recommended to take prescribed antibiotics for 2 weeks. Three weeks later, the patient came back without any improvement in his symptoms. Rechecking the PSA level, the result showed 435.1 ng/mL. With the initiative diagnosis of prostate cancer, a bone scan and prostate biopsy (under the guidance of transurethral ultrasonography) were requested. The whole‐body bone scan showed multiple focal areas of abnormally increased radiotracer uptake in the right clavicle and shoulder, right scapula, sternum, several ribs, pelvic bones, femur, many vertebral bodies, and skull.

**FIGURE 1:**
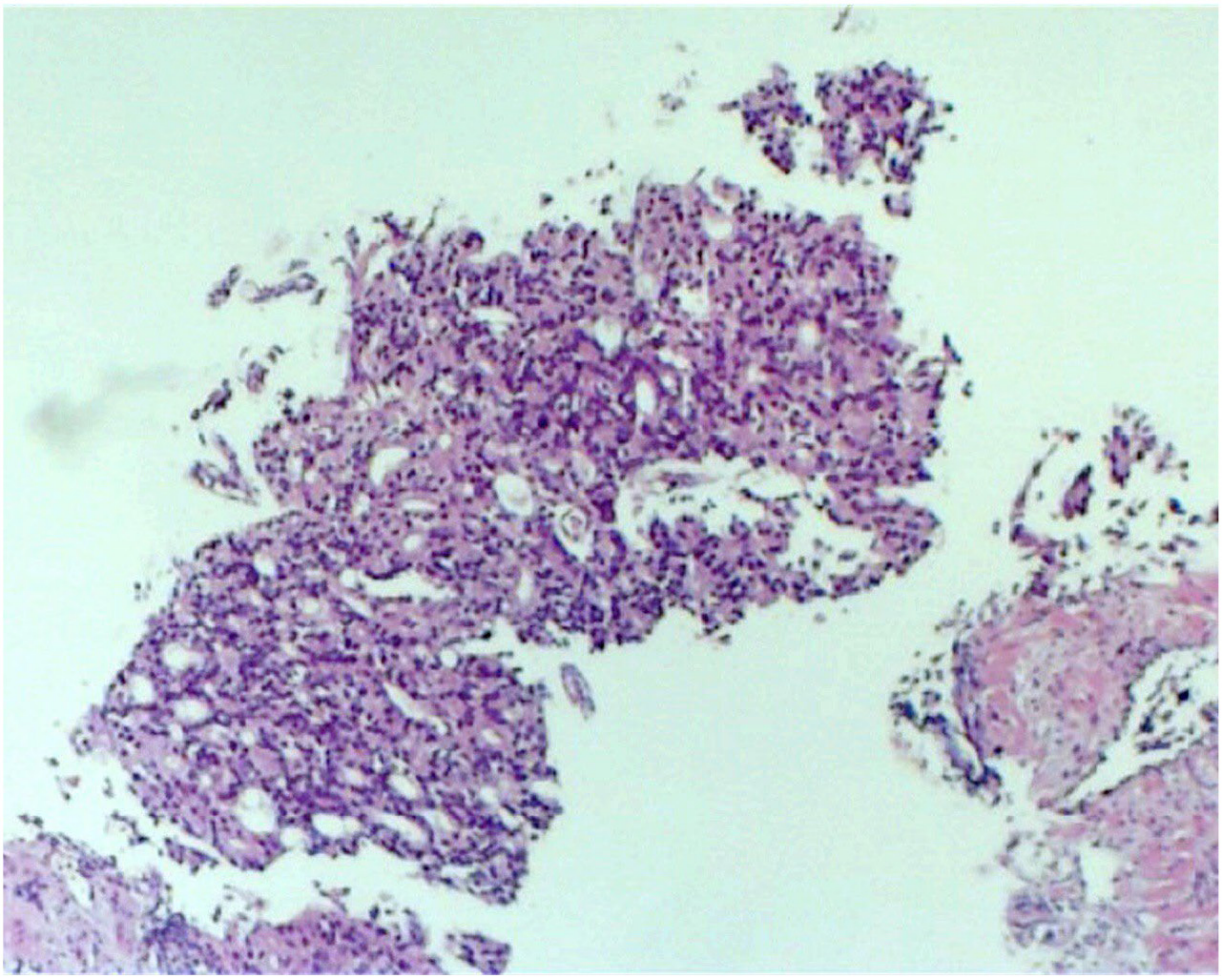
Prostate biopsy, adenocarcinoma (optical microscopy, hematoxylin, and eosin staining, 100× magnification).

**FIGURE 2:**
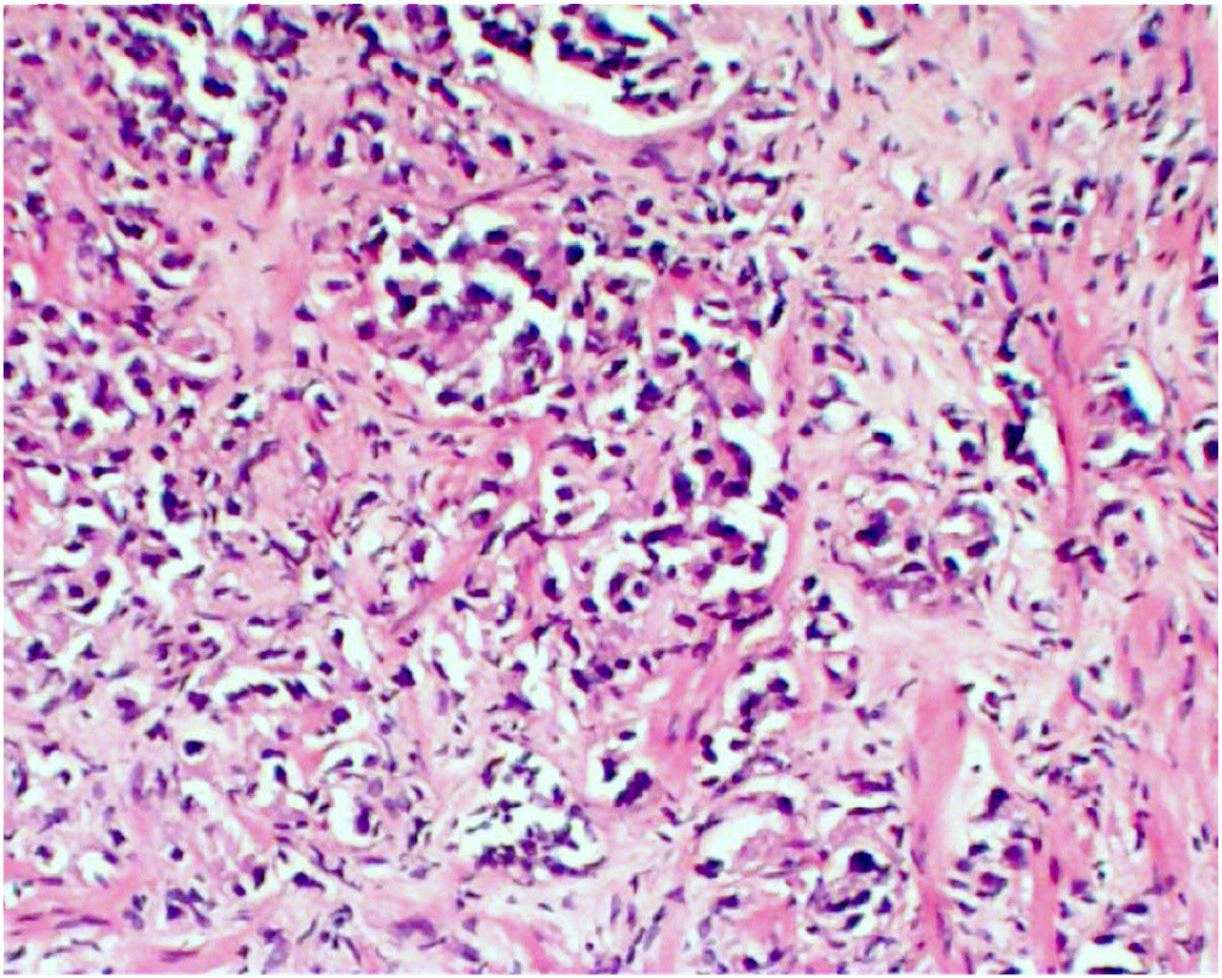
Prostate biopsy, adenocarcinoma (optical microscopy, hematoxylin and eosin staining, 200× magnification).

**FIGURE 3:**
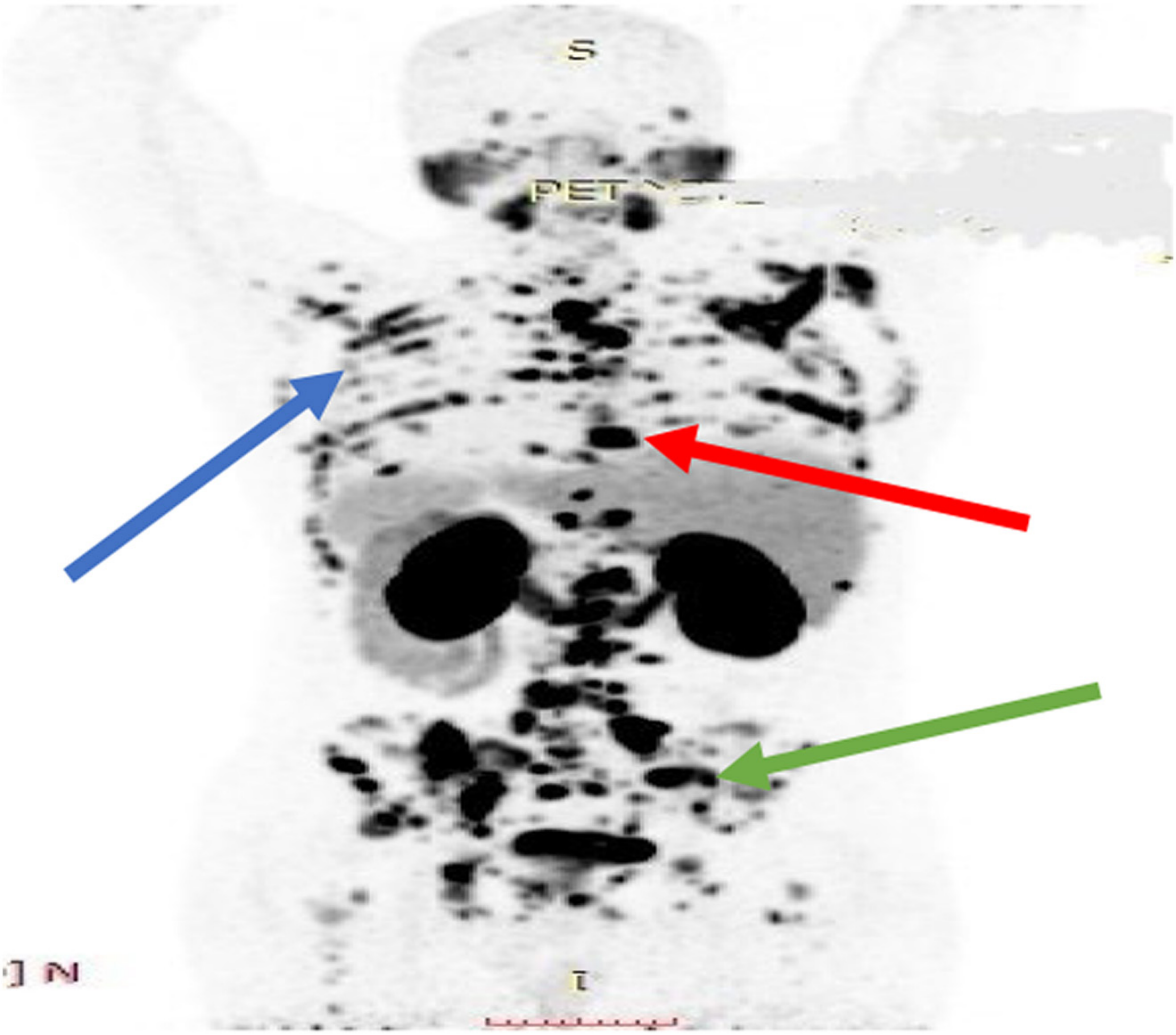
Positron emission tomography (PET) scan, several **(**PSMA)‐positive pelvic lymph nodes (green arrow) along with numerous skeletal lesions in the vertebrae (red arrow), ribs, and scapula (blue arrow).

The differential diagnoses considered were metastatic cancer and multiple myeloma. Ultrasonography of the prostate showed an enlargement measuring 45 mL in volume. Further laboratory investigations were suggestive of high alkaline phosphatase levels (146 U/L, normal range: 30–120) and normal calcium, phosphorus, 25(OH)vitamin D3, and parathyroid hormone levels, excluding bone diseases. The prostate biopsy results confirmed prostate adenocarcinoma in all 12 samples taken from the prostate. Gleason Scores: a range between 4/5 + 3/5 = 7/10 and 5/5 + 5/5 = 10/10 was reported (Figures 1 and 2). The percentage of involvement with tumors in samples varied from 10% up to 90%. Considering the pathology results, a pelvic CT scan and a positron emission tomography (PET) (Figure 3) scan were ordered, which showed several prostate‐specific membrane antigens (PSMA)‐positive pelvic lymph nodes (green arrow) along with numerous skeletal lesions in the vertebrae (red arrow), ribs, and scapula (blue arrow). The final diagnosis was advanced metastatic prostate adenocarcinoma with multiple skeletal and lymph node metastases. Because of multiple metastatic lesions, the patient's treatment started with Docetaxel and Zoladex (LHRH analogous) every 3 months and testosterone‐suppressing tablets (Abiraterone) 1 gram daily. The patient's ambulation and pain have improved significantly after the initiation of the treatment. The progression and improvement of the condition have been regularly monitored clinically, along with laboratory reports and imaging. The reevaluation by the neurologist was requested for the initial diagnosis of multiple sclerosis by referral of the patient. It revealed that the patient had no signs or symptoms of this disease, and there were no indications for further evaluation.

## 3 DISCUSSION

Prostate cancer is one of the two common causes of cancer in men and the overall fifth‐leading cause of death among both genders. The genetic component of prostate cancer is demonstrated by several studies, such as epidemiological studies, twin studies, and large‐scale genome‐wide association studies (GWAS).^12^, ^13^, ^14^, ^15^ Also, aging correlates directly with the incidence and mortality of prostate cancer worldwide. The average age of diagnosis is reported to be 66 years old. The joint regression analysis showed a significantly increased rate of incidence in all age groups, in contrast with declining mortality rates in these age groups over the past three decades. Prostate cancer's incidence rises significantly from 40‐ to 44‐year‐old men (less than 1 in 100,000 men) to the 45‐ to 49‐year‐old age group (more than 3 in 100,000 men). Several studies showed men in the age range of 40 and 79 years old have an increasing trend in both incidence and mortality with advancing age.^16^

There are several steps in the process of malignant transformation from a normal prostate to metastatic cancer, initiated as prostatic intraepithelial neoplasia (PIN), followed by localized prostate cancer, and then advanced prostate adenocarcinoma with local invasion, resulting in metastatic prostate cancer.^17^ In contrast with the good prognosis and high long‐term survival of prostate cancer localized to the gland, metastatic prostate cancer is mostly incurable despite treatments that approach several different targets.^18^ The main cause of death due to prostate cancer is metastasis and involvement of other organs. The first and most common metastasis site is regional lymph nodes.^19^ Other common sites of metastasis include the liver, lungs, and bones, which cause osteoblastic mixed with osteolytic lesions. Bone metastasis can cause hypercalcemia, frequent fractures of the bones, and severe pain.^18^

Early‐onset prostate cancer seems to be underestimated. However, recently, due to more common screening and increasing prevalence, about 10 percent of diagnosed prostate cancer is in men ≤55 years old. The main reason for changing the epidemiology of prostate cancer toward incidence in younger age groups can be attributed to PSA‐based screening.^2^ Evidence of this trend is rare reports of metastatic prostate cancer in younger men, such as the 40‐year‐old man with testicular metastasis of prostate cancer^20^ and also a 28‐year‐old man with metastatic prostate cancer with involvement of pelvic lymph nodes.^2^ However, the case presented in this report demonstrated unprecedented, vastly metastasized prostate cancer to vertebral and shoulder bones with a significantly high level of PSA. The important point in this case is the manifestation of the disease, which was mainly upper and lower limb bone pain and walking difficulty without significant genitourinary symptoms. Although Sahil Gupta et al. believe PSA levels cannot be a good sign of prostate cancer in men younger than 50 years old due to poorly differentiated adenocarcinoma of the prostate,^2^ this case showed that even in patients younger than 50 years old, PSA can be one of the means of diagnosis, determining the progression and prognosis of prostate cancer. Ethan M. Lange et al.'s findings demonstrated the more important role of genetic factors in the occurrence of early‐onset prostate cancer in comparison with later‐onset cases.^20^, ^21^

The management of patients with prostate cancer differs based on the extent of the disease, the patient's condition, and their age. For older patients with a lower life expectancy, the wait‐and‐watch method seems appropriate. However, younger patients without involvement of other parts of the body are mainly treated with radical prostatectomy if they wish and have the conditions appropriate for surgery. In contrast, those with involvement in lymph nodes that are detected on imaging should be treated with androgen deprivation therapy (ADT). This can be done either with bilateral orchiectomy or medication (such as Zoladex in this patient). New drugs, many of which are informed by different genomic pathways, are under development. Parallel to the recent development of locoregional therapies for metastatic cancers, patients' conditions are managed more effectively by applying existing therapeutic options.^22^ Two‐year follow‐up imaging and laboratory data showed our patient was appropriately managed with ADT and the disease was controlled despite advanced metastasis.

## 4 CONCLUSION

Prostate cancer's initial manifestation might be completely distinct from genitourinary system symptoms or with unusual presentations in this system as reported in previous literature (Table 1). These patients might be wrongly referred to neurologists and orthopedic surgeons. Moreover, prostate cancer should not be excluded in younger patients with symptoms alarming this disease. Although PSA might not be an excellent indicator and screening test for highly probable prostate cancers due to lower levels of this marker secreted by poorly differentiated prostate cells, considering available screening options, this specific antigen seems to be the best screening tool for prostate cancer, even in younger patients.

**TABLE 1:** Uncommon presentations of prostate cancer in other literature.

Article and authors	Age	Presentation	Treatment/Progression or remission of the disease
Unusual presentations of prostate cancer: A review and case reports Elabbady et al.^8^	57‐year‐old	Right hard supraclavicular mass/2 months later by aphasia and right‐sided hemiparesis/metastatic brain tumors affecting the left temporal, parietal, and frontal lobes/no urinary complaints/PSA:151 ng/m/diffuse skeletal metastases/gleason 4 + 5 in all four cores	Bicalutamide 150 mg daily + corticosteroids/LHRH analogs/followed up for >18 months, on intermittent androgen blockade/on intermittent androgen blockade, and showing stable disease
Unusual presentations of prostate cancer: A review and case reports Elabbady et al.^8^	77‐year‐old	History of BPH/acute urinary retention/hard, irregular prostate in DRE/PSA: 180 ng/mL/no skeletal metastases and no abdominal lymphadenopathy/Gleason 4 + 5 in all six cores taken/bicalutamide 50 mg/day	Bicalutamide 50 mg/dayLHRH agonist/After 1 year of controlled disease the patient was followed while on intermittent androgen blockade/good PSA response (reduced to 0.09 ng/mL)/during the off‐treatment period the patient presented with a large, hard, and fixed left supraclavicular lymph node, with a PSA level of 12 ng/mL/complete resolution of the supraclavicular mass within 3 months of androgen blockade
Cutaneous metastasis of prostate cancer: a case report and review of the literature with bioinformatics analysis of multiple healthcare delivery networks Brown et al.^7^	73‐year‐old	Persistent enlarging rash involving the left chest 4 years after diagnosis of prostate cancer/purpuric papula‐nodular plaque with surface excoriations and ecchymotic lines, PSA: 400 ng/mL/Gleason score of 7 (3 + 4)	Metastasis to lymph nodes and bones/died in 1 year despite receiving chemotherapy
Brain metastasis from prostate cancer: A case report Zhang et al.^9^	72‐year‐old	Difficulty in urination/enlarged, firm prostate gland with irregular nodules/PSA: 40.6 ng/mL/2 years later: transient attack of unconsciousness due to brain metastasis/intracerebral mass measuring 2 cm × 2 cm in right frontal lobe/additional multiple metastases were found in the spine and lung	Transurethral prostatectomy for the remission of his difficult voiding/2 g ifosfamide per day/local radiation therapy/2 years later: transient attack of unconsciousness due to brain metastasis/surgically resection of brain tumor/500 mg estramustine (Estracyt) per day. Unfortunately, the patient died of pneumonia 19 months after the diagnosis of brain metastasis
Metastatic prostate cancer presents as an asymptomatic neck mass Carleton et al.^11^	84‐years‐old	Diagnosed with prostate cancer 15 years ago with a left supraclavicular mass/increasing swelling on the left side of his neck/a non‐tender left‐sided neck mass roughly 4 cm in diameter/a 4.2 cm, solid left supraclavicular mass with extension from the clavicles to the true vocal cords/PSA 55.5 ng/mL	Radical retropubic prostatectomy and bilateral pelvic lymph node dissection 15 years ago/total androgen ablation with leuprolide (Lupron) and bicalutamide (Casodex)/biopsy of the left supraclavicular: prostate adenocarcinoma/the patient declined additional therapy such as secondary hormonal manipulation, systemic chemotherapy, and radiation for localized disease
Prostate cancer: Cases of rare presentation and rare metastasis Hiren Mandaliya et al.^23^	80‐year‐old	Diagnosed with prostate cancer 4 years ago/with painless swelling in his right breast. It was a 5 × 4 cm sized, hard, non‐tender swelling/no lymphadenopathy or bone tenderness/lobulated retro‐areolar lesion of the breast measuring 18 × 20 × 46 mm in diameter with well‐defined margins, more in Favor of atypical gynecomastia in US/ipsilateral axillary or supraclavicular or neck lymphadenopathy/core biopsy showed a malignant infiltrate with positive staining for PSA	On leuprorelin and bicalutamide from the initial diagnosis/the treatment and the future follow‐up of the patient have not been stated in the paper
Testicular metastasis of prostate cancer: A case report Kusaka et al.^26^	56‐year‐old	PSA: 137/palpable prostate with hard consistency on both lobes/Gleason score of 9/multiple lymph node enlargements from the pelvis to the bifurcation of the left renal artery on CT/multiple skeletal metastases in the left ilium and ischium in bone scan/stage IV (T3N1M1b) prostate cancer/right testicular swelling 4 months later/solid yellowish‐white tumor of 5 × 4 cm/Gleason score of 8	Androgen deprivation therapy (ADT) for 4 years, followed by luteinizing, hormone‐releasing hormone, and estramustine for 1 year/radiation therapy to the prostate/despite ADT, his serum PSA level increased/right high inguinal orchiectomy was performed/has been asymptomatic, disease‐free/PSA level decreased to undetectable level in 1 year
Malignant priapism secondary to metastatic prostate cancer: A case report and review of literature Lin et al.^10^	84‐year‐old	With the previous diagnosis of prostate cancer presented/a 3‐month history of persistent erection since he finished radiation treatment/Gleason 4 + 5/sharp and burning pain that was only mildly relieved by topical lidocaine gel/possible partial cavernosal thrombosis at the base of the cavernosal as well as plaques within the tunica albuginea in MRI/rigid penile shaft and glans, with pain on palpation/decreased sensation along the shaft. extensive amount of fibrosis and necrosis of each of the cavernosal bodies in cavernostomy/biopsy: adenocarcinoma of cancer/metastatic disease in left lower lung, liver, and abdominal and pelvic lymph nodes	Radiation treatment at initial diagnosis/detumescence in the past 6 weeks and phenylephrine but no response to treatment/surgical repair of priapism and penile exploration/consultation according to the extent of disease and treatment options/referral to oncology for treatment and palliative care
Mandibular metastasis of adenocarcinoma from prostate cancer: case report according to epidemiology and current therapeutical trends of the advanced prostate cancer Menezes et al.^24^	54 years old	Pain in the left mandibular region above the median line/paresthesia of the anatomical areas sensitized by the left alveolar/inferior nerve, a branch of the mandibular nerve and trigeminal nerve/after a mandibular abscess 1 year before/left mandibular bulging in the buccolingual aspect and tooth mobility involving bulging region extending to the opposite canine tooth and a slight gums erythema/anatomical structure alteration with osteolysis areas in the premolars, molars and in the left region of the chin in CT‐scan/adenocarcinoma (clear cell type) with necrosis areas and infiltrating bone and soft tissue in incisional biopsy/mandibular region, where increased uptake of the radiocontrast agent as in other areas, such as the right clavicle and scapula, humerus, ribs, spine, sternum, hip bone, and both femurs	Initial diagnosis of Trigeminal Neuralgia/treatment with Carbamazepine Gabapentin/radical prostatectomy performed 1 year after prostate cancer diagnosis/good disease control, good tolerance to treatment for a few months following goserelin acetate (3.6 mg/month) and bisphosphonate (clodronate, 4 ampoules/month)/patient's death in less than a year by disseminated neoplasm and pulmonary involvement
Prostate Cancer Presenting as Hip Pain at the Chiropractic Office: A Case Report and Literature Review Chu et al.^25^	62‐year‐old	Seven‐day history of deep and aching pain in the front of the left groin region radiating down the thigh to the knee/range of motion of the left hip was limited due to pain/flexion abduction external rotation (FABER) of the hip and sacroiliac joint spring tests reproduced pain in the left hip, lumbosacral spine, and sacroiliac joint/strength 4/5 in left hip flexion/hypertonicity at the paraspinal muscles, gluteal muscles, iliotibial band, lateral rotators, and adductors of the left hip in palpation/after partial responsiveness to the treatment of chiropractic referred to an oncologist and in PET‐scan, PSA, and biopsy/mixed lytic sclerotic lesions of medial wall of the left acetabulum and left ischium with Cortical destruction and soft tissue penetration/a hypermetabolic prostatic lesion was observed involving the right half of the prostate/diagnosis of lung and lymph node metastasis Numerous sub‐centimeter nodules were scattered in both lungs	Before prostate cancer diagnosis: With the initial diagnosis of soft tissue injury of the hip joint and was treated with non‐steroidal anti‐inflammatory medication and sports rehabilitation without further investigation by his primary care physician due to hip pain after marathon After prostate cancer diagnosis: androgen deprivation, novel hormonal agents, systemic chemotherapy, and bone resorptive agents/continued chiropractic therapies, hip rehabilitation home exercises/7 months after prostate cancer diagnosis by biopsy and partial improvement of pain died by a lung infection

## AUTHOR CONTRIBUTIONS

**Homa Taheri:** Investigation; resources; software; supervision; validation; writing – review and editing. **Pouya Ebrahimi:** Conceptualization; data curation; methodology; project administration; resources; supervision; writing – original draft. **Pedram Nazari:** Formal analysis; project administration; writing – review and editing. **Amirhossein Kefayat:** Validation; writing – review and editing. **Abbas Mahdavian:** Data curation; formal analysis; supervision.

## CONSENT

Written informed consent was obtained from the patient to publish this report in accordance with the journal's patient consent policy.

## Data Availability

Data openly available in a public repository that issues datasets with DOIs.
